# Social Role–Based Training Using a Computer Graphics Avatar in a Real-World Commercial Setting for Individuals With Autism Spectrum Disorder: Quasi-Experimental Study

**DOI:** 10.2196/93549

**Published:** 2026-09-30

**Authors:** Hirokazu Kumazaki, Hiroko Kamide, Takahiro Miyashita, Hidenobu Sumioka, Yuichiro Yoshikawa, Hiroshi Ishiguro

**Affiliations:** 1 Department of Neuropsychiatry Graduate School of Biomedical Sciences Nagasaki University Nagasaki Japan; 2 Kanazawa University Kanazawa, Ishikawa Japan; 3 The Center for Interdisciplinary Studies of Law and Policy Kyoto University Kyoto, Kyoto Japan; 4 Interaction Science Laboratories Advanced Telecommunications Research Institute International Souraku, Kyoto Japan; 5 Department of Systems Innovation Graduate School of Engineering Science The University of Osaka Toyonaka, Osaka Japan

**Keywords:** ASD, autism spectrum disorder, computer graphics avatar, real-world interactions, self-efficacy, social role–based behaviors

## Abstract

**Background:**

The lack of social role–based behaviors, such as guiding and assisting others, is a barrier to employment success and community participation for individuals with autism spectrum disorder (ASD). Enhancing self-efficacy is essential for improving these behaviors. To enhance self-efficacy in social role–based behaviors, we developed a training system that enables users to perform social role–based behaviors, including guiding and assisting others, called TCR (a training system that enables users to perform role-based behaviors using a computer graphics avatar in a real, bustling commercial facility).

**Objective:**

We aimed to evaluate the feasibility of TCR and its potential to reduce anxiety and enhance self-confidence, motivation, metacognition, and self-efficacy in performing social role–based behaviors during real-world interactions among individuals with ASD.

**Methods:**

We assigned 38 adults with ASD to either the TCR group (n=20), which received TCR alongside communication guidance from teachers (CGT), or the control group (n=18), which received only CGT, based on the chronological order of enrollment. Both groups underwent a 5-session intervention (days 14, 21, 28, 35, and 42). Anxiety, self-confidence, motivation, metacognition, and self-efficacy related to social role–based behaviors were assessed on days 0, 14, 28, 42, and 56 using self-report and observer ratings.

**Results:**

Linear mixed model analyses showed group × time interactions for self-reported self-efficacy at day 56 (*t*_144.016_=2.670; *P*=.008; 95% CI 0.349-2.340). Nominally significant group × time interactions were also observed for self-reported anxiety, self-confidence, motivation, and metacognition, suggesting greater improvements in the TCR group than in the control group. Observer-reported outcomes demonstrated significant group × time interactions for self-efficacy at day 56 (*t*_144.016_=4.500; *P*<.001; 95% CI 0.405-1.039), anxiety at day 56 (*t*_144.016_=–3.593; *P*<.001; 95% CI –1.425 to –0.414), and self-confidence at day 56 (*t*_144.016_=3.321; *P*=.001; 95% CI 0.269-1.059), indicating greater improvement in the TCR group than in the control group. Nominally significant group × time interactions were additionally observed for observer-reported motivation at day 56 (*t*_144.016_=2.471; *P*=.02; 95% CI 0.103-0.930). Possible group × time interactions were also observed for observer-reported metacognition at day 56 (*t*_144.016_=1.858; *P*=.06; 95% CI –0.023 to 0.751).

**Conclusions:**

This study is innovative in that TCR enables users to perform social role–based behaviors. Unlike previous studies, TCR integrates opportunities for observing peers and role models, along with real-time encouragement, feedback, and verbal persuasion from a trainer, thereby incorporating multiple sources of self-efficacy. By demonstrating the feasibility of TCR and its potential to enhance self-efficacy in social role–based behaviors, this study contributes to the development of more effective interventions targeting functional social outcomes in individuals with ASD. TCR may support the development of skills relevant to employment and social participation in real-world settings and represents a potential scalable approach for community-based ASD interventions.

## Introduction

Autism spectrum disorder (ASD) comprises a range of conditions categorized by challenges in social skills, repetitive behaviors, and verbal and nonverbal communication. The United States Centers for Disease Control and Prevention reports that approximately 1 in 36 children has ASD [1]. While most studies so far have focused on children, ASD in adults has become a major social concern in recent years. Employment is one of the most desirable achievements for individuals with ASD when they enter adulthood. However, many adults with ASD experience poor vocational outcomes [2-5]. A lack of social role–based behaviors, including guiding and assisting others, is a major barrier to employment success and community participation in individuals with ASD [6,7]. Enhancing self-efficacy is essential for improving social role–based behaviors [8]. However, interventions aimed at enhancing self-efficacy in social role–based behaviors have not yet been established.

Self-efficacy was originally introduced by Bandura [9] as a central concept in his social learning theory (later called social cognitive theory), and this concept has been supported in subsequent research [10-12]. He identified 4 main sources (elements) that shape and strengthen self-efficacy beliefs: mastery experiences (successfully completing tasks or overcoming challenges builds confidence in one’s abilities), vicarious experiences (observing others, especially peers or role models, and succeeding through sustained effort), verbal persuasion (encouragement, feedback, and persuasion from others that one can succeed), and physiological and affective states (one’s physical and emotional condition affects perceived ability; high stress, fatigue, or anxiety can lower self-efficacy, while positive mood and good physical state can enhance it). These elements, along with structured feedback, appropriately graded challenges, and peer support, play essential roles in strengthening individuals’ self-efficacy in social role–based behaviors.

Given that individuals with ASD often accumulate negative social experiences, mastery experiences may reduce avoidance behavior, decrease social anxiety, and promote engagement. Vicarious experiences may facilitate social learning by providing observable models of appropriate social behaviors, thereby enhancing social self-efficacy. Verbal persuasion may support social interactions by offering explicit guidance and reinforcement, which is particularly important given difficulties in interpreting implicit social cues. Finally, physiological and affective states may strongly influence social interactions, as heightened anxiety and dysregulated arousal can negatively impact perceived self-efficacy and lead to avoidance. Interventions that reduce anxiety and promote adaptive interpretations of internal states may therefore facilitate social engagement. These elements, along with structured feedback, appropriately graded challenges, and peer support, play essential roles in strengthening individuals’ self-efficacy in social role–based behaviors.

Many individuals with ASD have difficulty sustaining motivation and concentration during interactions with people in real-world social settings [13]. According to the “intense world theory” [14], individuals with ASD may perceive their surroundings as overwhelmingly intense due to hyperreactivity of primary sensory areas and as aversive and highly stressful due to an overly reactive amygdala—factors that contribute to heightened social anxiety. This heightened sensory processing may be triggered by dynamic facial features, particularly direct gaze and facial expressions, which can induce sensory and emotional overstimulation and lead to distraction [15]. Individuals with ASD may resort to social avoidance to cope with aversive overstimulation. Reducing social anxiety is a promising approach for promoting social engagement among individuals with ASD. Social motivation, which varies considerably across the autism spectrum, plays a key role in fostering smooth interpersonal relationships and coordination. Social communication interventions are most effective when they involve activities and settings that are intrinsically motivating [13]. Additionally, greater self-confidence is important for tackling real-world situations and social encounters [16,17]. Taken together, interventions that simultaneously address social anxiety, enhance social motivation, and foster self-confidence may be particularly effective in improving social role–based behaviors among individuals with ASD.

Many studies have reported reduced metacognitive accuracy in individuals with autism compared with those without, although performance remains heterogeneous and not all individuals show such difficulties [18,19]. Moreover, metacognition has been shown to mediate the effects of social communication skills training in individuals with ASD [20], suggesting that enhancing metacognitive abilities may help improve social role–based behaviors.

Recent advances in technology, such as virtual reality (VR), mobile apps, interactive software, and screen-based systems, have provided less stressful alternatives for individuals with ASD, helping to reduce the anxiety often associated with practicing real-world social communication. In particular, VR-based interventions are effective in enabling individuals to gain successful experiences in a secure setting while reducing stress. Several VR programs have focused on improving social role–based behaviors [21-28]. While some studies have indicated that individuals with ASD can understand, use, and respond effectively to virtual environments and may transfer these skills to real-world settings [21,26-28], generalization of these findings remains a major concern. For instance, Astafeva et al [27] developed a VR system that simulates complex real-world environments, whereas Alvari et al [28] developed a system designed to adapt to the user’s anxiety level. Users may struggle to maintain newly acquired social role–based behaviors in real-life contexts long after the intervention [29,30]. Given that individuals with ASD often face challenges in flexible thinking and generalization [31-33], it is not surprising that transferring skills from conventional therapies to real-world situations can be difficult. Therefore, learning environments that incorporate real interpersonal interactions in safe, real-world contexts could be particularly beneficial for helping individuals with ASD generalize their social role–based behaviors.

Individuals with ASD often exhibit intrinsic motivation when using teleoperated devices [34-37]. For individuals with ASD who feel anxious about face-to-face communication, an intervention that allows them to operate an avatar to interact with others can be beneficial, as their interlocutors cannot see them directly. This could enable them to avoid eye contact while engaging in real-time verbal and nonverbal exchanges through the avatar and help them overcome challenges and accomplish their tasks. Another advantage is that individuals with ASD can observe the facial expressions and features of their interlocutors at their own pace. These interventions integrate the elements of mastery experiences and physiological and affective states as conceptualized in social learning theory by Bandura [9]. Kanchi et al [37] showed that an intervention using an avatar reduced anxiety and enhanced the self-confidence and motivation of individuals with ASD to communicate in a real-world setting.

With a goal of enhancing self-efficacy in social role–based behaviors, in this study, we developed a training system that enables users to perform role-based behaviors, including guiding and assisting others, using a computer graphics (CG) avatar in a real, bustling commercial facility (TCR: a training system that enables users to perform role-based behaviors using a computer graphics avatar in a real, bustling commercial facility). We aimed to evaluate the feasibility of TCR and its potential to reduce anxiety and enhance self-confidence, motivation, metacognition, and self-efficacy in performing social role–based behaviors during real-world interactions among individuals with ASD.

## Methods

### Ethical Considerations

This study involving human participants was reviewed and approved by the Ethics Committee of Kanazawa University (approval number 2096-12) and was conducted in accordance with the Declaration of Helsinki. Following a comprehensive explanation of the study, written informed consent was obtained from all participants prior to study participation. All data were deidentified prior to analysis to protect participant privacy and confidentiality. Participants did not receive any financial compensation for participating in the study. No images included in the manuscript or supplementary materials contain identifiable participant information.

### Inclusion and Exclusion Criteria

The inclusion criteria were as follows: (1) a diagnosis of ASD based on the *DSM-5* (*Diagnostic and Statistical Manual of Mental Disorders [Fifth Edition]*), (2) being aged 18 years or older, and (3) not taking any medication. At the time of enrollment, the diagnoses of all participants were confirmed by a psychiatrist with more than 20 years of experience in ASD using the *DSM-5* criteria and the Diagnostic Interview for Social and Communication Disorders (DISCO) [38], which exhibits good psychometric properties [39]. Participants were excluded if they had intellectual disability, comorbid attention-deficit/hyperactivity disorder (ADHD), or were unable to communicate verbally.

### Participant Characteristics

All participants were adults with ASD, without intellectual disability, and were able to communicate verbally. Detailed demographic and clinical characteristics are presented in the Results section.

### Sampling Procedures

Participants were recruited from a psychosocial rehabilitation facility for adults with ASD in Japan that specializes in developmental disorders and related conditions. A recruitment notice describing the study was posted on the facility bulletin board. Individuals who expressed interest in participating were screened for eligibility according to the inclusion and exclusion criteria. Participation was voluntary, and all participants independently decided to take part in the study. Recruitment was conducted between July 2025 and October 2025.

### Sample Size, Power, and Precision

A total of 38 participants were enrolled in this study. No a priori power analysis was conducted because of the exploratory nature of this study. The sample size was determined based on participant availability and feasibility considerations.

### Conditions and Design

This study used a quasi-experimental design involving an experimental manipulation without participant randomization. The unit of assignment was the individual participant. Participants were assigned to the intervention or control group according to the chronological order of enrollment, without randomization. No restrictions on assignment, such as blocking or stratification, were applied. Furthermore, no procedures, including matching or propensity score methods, were used to minimize selection bias. Participants in the intervention group received TCR alongside communication guidance from teachers (CGT), whereas those in the control group received CGT alone. The intervention consisted of 5 weekly sessions. Outcomes were assessed to evaluate changes associated with the intervention.

### Masking

Masking was not implemented because of the nature of the intervention. Participants, teachers administering the intervention, and outcome assessors were aware of group assignments.

### Procedure

In this study, participants were assigned to 1 of 2 groups: the TCR group (receiving TCR alongside CGT in a classroom setting) or the control group (receiving only CGT) based on the chronological order of enrollment (Figure 1). For CGT, a single teacher conducted classes in one-on-one sessions using an original textbook with role-playing scenarios on guiding and assisting others in social contexts, and participants held discussions with teachers based on these scenarios.

**Figure 1:**
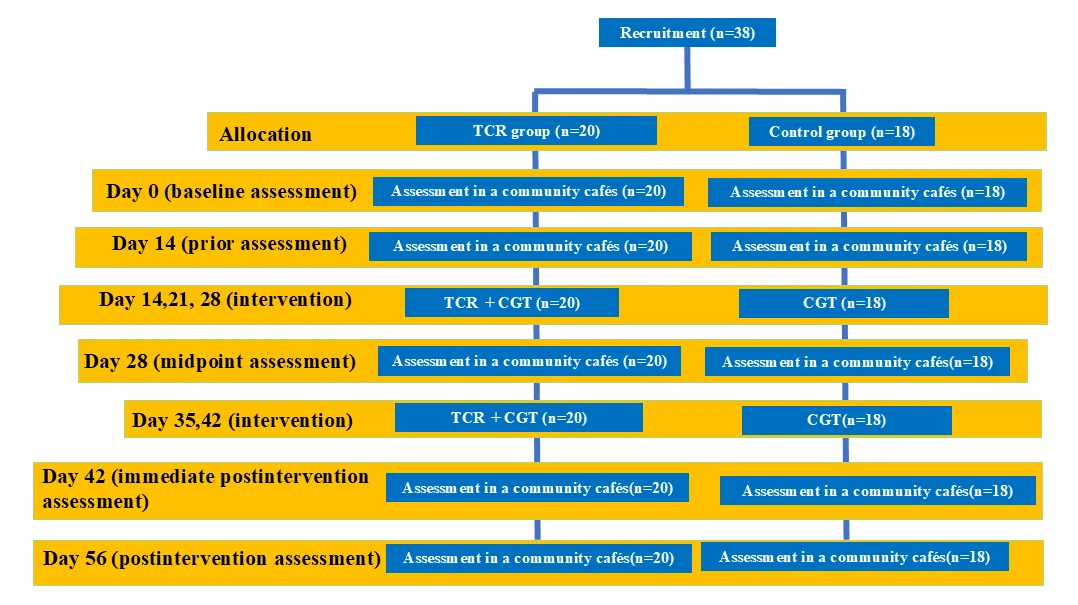
Study flow and assessment schedule. This was a nonrandomized controlled study. All participants had autism spectrum disorder. The study was conducted in a psychosocial rehabilitation facility. Participants were assigned to 1 of 2 groups: the TCR (a training system that enables users to perform role-based behaviors using a computer graphics avatar in a real, bustling commercial facility) group (n=20, received TCR alongside communication guidance from teachers [CGT] in a classroom setting) and the control group (n=18, received only CGT) based on the chronological order of enrollment. Both groups underwent a 5-day intervention. The experimental procedures were performed from days 0 to 56. The interventions were conducted once per week for 5 weeks (days 14, 21, 28, 35, and 42). During the intervention period, the control group received only CGT, whereas the TCR group received TCR training and CGT. Anxiety, self-confidence, motivation, metacognition, and self-efficacy were assessed in a real-world setting at 5 time points: before the intervention (day 0), immediately before the intervention (day 14), during the intervention (day 28), immediately after the intervention (day 42), and 2 weeks after the intervention (day 56). There were no missing data at either the participant (0%) or the item level (0%) in this study. All enrolled participants (n=38) were included in the final analysis, and no attrition or loss to follow-up occurred.

The interventions were conducted once per week for 5 weeks (days 14, 21, 28, 35, and 42). During the intervention, the control group received only CGT (1 hour per day), whereas the TCR group received both TCR and CGT (each approximately 1 hour per day). TCR and CGT were conducted in a psychosocial rehabilitation facility. No additional homework or between-session assignments were provided. We created a real-world setting in which participants performed role-based behaviors to evaluate whether the intervention could be generalized to real-world situations.

### Data Collection

This setting consisted of community cafés in which unfamiliar individuals were present. The participants were instructed to perform role-based behaviors, including guiding and assisting others at the community café. Observations were made in real time without using video recordings. No scripts were provided to the participants. Unfamiliar individuals at the café were randomly selected as visitors on the day of assessment. Besides informing the visitors in advance that they would interact with individuals participating in a social communication rehabilitation program for ASD, no modifications were made to the environment. All assessments lasted for approximately 1 hour, during which assessors evaluated participants’ behaviors based on their interactions with guests. We assessed participants’ anxiety, self-confidence, social motivation, metacognition, and self-efficacy regarding social role–based behaviors. Each interpersonal communication session lasted approximately 1 hour. Approximately 10 people visited the café every hour. These assessments were conducted 2 weeks before the intervention (day 0), immediately before the intervention (day 14), during the intervention (day 28), immediately after the intervention (day 42), and 2 weeks after the intervention (day 56) to evaluate its generalizability in real-world situations.

### Instrument

The teleoperated avatar used in this study was the CG agent of AVACOM (manufactured by AVITA), which is a cartoon-style CG agent with a neutral appearance and generic design. This system was developed to provide participants with a rich sense of presence through lifelike behaviors and fully customized contemporary anime-style expressions. It uses a full-body 3D model capable of producing cartoon-style images with lifelike expressions using facial and whole-body movements. Its formal appearance and rich motions enable it to guide others confidently. In this system, participants can play the role of a guide in a real, bustling commercial facility by simply pressing 4 buttons in numerical order.

In addition to button-based control, an avatar could be operated by converting a participant’s speech and physical movements into corresponding actions. When participants spoke through a microphone connected to a PC, the CG agent repeated the words. The participant’s voice was converted using a voice-conversion system to match the avatar’s appearance. Moreover, there was a motion-capturing system for the participant, allowing the CG avatar to reflect the participant’s gestures and enact a high degree of nonverbal communication.

In TCR, the participant is seated in front of a monitor with a device equipped with a small number of buttons, which is easy to use even for individuals with few digital skills. The CG avatar, placed in a real, bustling commercial facility, spoke when a button was pressed (Figure 2). When the participants were accustomed to pressing the button and the participant spoke into a microphone, the CG avatar would transmit the participant’s spoken words simultaneously. Thus, as the conversation progressed, the participant would shift from simple to complex procedures (ie, beginning by pressing a button and advancing to voluntary speech as the participant became accustomed). Through this setup, participants could perform role-based behaviors, such as guiding and assisting unfamiliar people in a bustling commercial environment, while remaining in their psychosocial rehabilitation facility, a familiar and comfortable setting physically separated from others. Thus, like other avatar-based systems, the TCR integrates the elements of mastery experiences and physiological and affective states, as conceptualized in social learning theory by Bandura [9], with a gradual increase in challenge levels. Moreover, in this system, participants with ASD were grouped in pairs: one in the role of the operator and one sitting next to the operator responsible for observing and evaluating the operator’s behavior. In other words, they had the opportunity to observe their peers and role models. By observing and evaluating the operator’s behavior during the intervention, participants may improve their ability to monitor and reflect on their own performance, thereby supporting metacognitive deficits. A trainer behind them (a licensed therapist who was not informed of the study objectives to minimize potential bias) provided encouragement, feedback, and persuasion to help the participants believe they could succeed after the intervention in a face-to-face setting.

**Figure 2:**
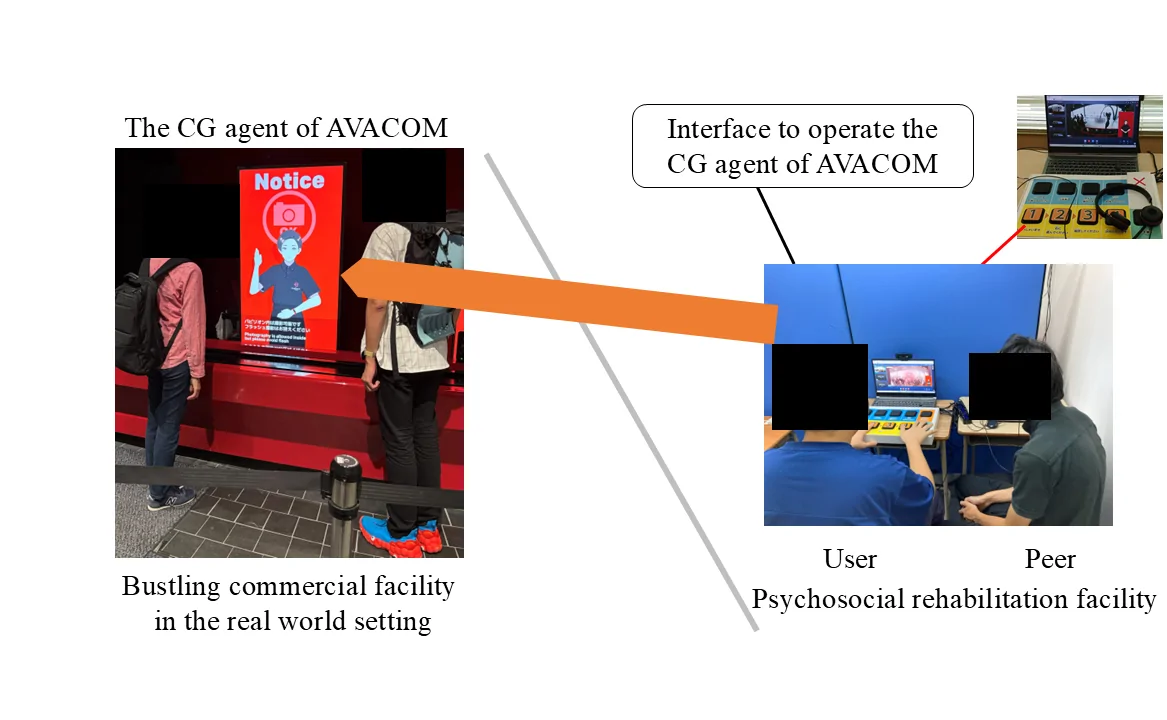
Illustration of the TCR (a training system that enables users to perform role-based behaviors using a computer graphics avatar in a real, bustling commercial facility) intervention setting. The user (right) pushes a button or speaks into a microphone connected to a PC that controls the computer graphics (CG) avatar (AVACOM) displayed in a real, bustling commercial facility. Using the avatar, the user can communicate with unfamiliar individuals in real time. A peer sitting next to the operator was responsible for observing and evaluating the operator’s behavior. A trainer behind them provided encouragement, feedback, and persuasion to help the participants believe they could succeed after the intervention.

Therefore, the system incorporates additional sources of self-efficacy, including vicarious experiences, verbal persuasion, clear feedback, and peer support. The participants assumed the roles of the operator and observer, and each role lasted approximately 30 minutes per day.

### Measures and Covariates

A set of standardized scales was used to characterize the participants’ profiles. The Japanese Adult Reading Test (JART) [40] is a standardized reading test used to estimate premorbid IQ in adults. Hidese et al [41] reported that JART scores significantly correlated with the IQ scores obtained from the Wechsler Adult Intelligence Scale, supporting the validity of the JART as an estimate of intellectual functioning.

Participants completed the Japanese version of the Autism-Spectrum Quotient–Japanese version (AQ-J) [42], which assesses behaviors and traits specific to ASD across cultures [43] and age groups [44-46] and is sensitive to broader autism phenotypes. In this study, we did not set a cutoff based on the AQ-J score and used only the *DSM-5* and DISCO to diagnose ASD and include participants in the study.

The Adolescent/Adult Sensory Profile (AASP) [47] is a self-administered questionnaire used to measure sensory processing in individuals aged 11 years or older. Its internal consistency coefficients range from 0.64 to 0.78 on the quadrant scores. Participants indicated how often they exhibited certain behaviors in relation to sensory experiences on a scale of 1 (“almost never”) to 5 (“almost always”). The AASP examines the 4 “quadrants” of sensory processing: low registration, sensation seeking, sensory sensitivity, and sensation avoidance. These quadrants are 2D and are not mutually exclusive. These dimensions help to form distinct profiles that reflect individuals’ sensory processing preferences and tendencies. Perceptual domain analysis was not performed in this study because the AASP does not classify responses according to individual “perceptual domains” (such as the auditory, visual, or tactile domains). The AASP was specifically evaluated because traits such as heightened sensory seeking could potentially influence the participants’ affinity for operating avatars.

The severity of participants’ social anxiety traits was measured using the Liebowitz Social Anxiety Scale (LSAS) [48]. This clinician-led scale comprises 24 items: 13 describing performance situations and 11 describing social interactions. Each item was rated separately for “fear” and “avoidance” on a 4-point categorical scale. Receiver operating characteristic analyses showed that an LSAS score of 30 was the optimal cutoff value for distinguishing between individuals with and without social anxiety disorders and correlated with minimal traits [49]. Previous studies [50,51] have supported the usefulness of the LSAS in assessing social anxiety in adults with ASD.

We developed a Likert scale specifically for this study to measure participants’ anxiety, self-confidence, motivation, metacognition, and self-efficacy levels during social role–based behaviors. Anxiety was assessed using a 7-point scale (1=no anxiety; 7=extreme anxiety). Self-confidence was assessed using a 7-point scale (1=very low confidence; 7=very high confidence). Motivation was assessed using a 7-point scale (1=not motivated at all; 7=highly motivated). Metacognition was assessed using a 7-point scale (1=very low; 7=very high). Self-efficacy was assessed using a 7-point scale (1=not confident that I could perform this behavior successfully; 7=extremely confident that I could perform it successfully). Self-confidence was conceptualized as a general subjective sense of confidence in engaging in social role–based behaviors. In contrast, self-efficacy refers to individuals’ beliefs about their capability to successfully perform these behaviors.

Anxiety, self-confidence, motivation, metacognition, and self-efficacy related to performing social role–based behaviors were assessed in all participants in the TCR and control groups on days 0, 14, 28, 42, and 56 by the participants themselves and 2 research assistants who were unaware of the study’s objectives.

The participants’ impressions of the appearance of the CG agent of AVACOM were assessed using a 7-point Likert scale in a self-reported questionnaire (1=not at all socially desirable; 7=extremely socially desirable) on day 42. The participants’ perceived usefulness of the experience for observing and evaluating the operator’s behavior was assessed using a 7-point Likert scale in a self-reported questionnaire (1=not useful at all; 7=extremely useful) on day 42. No covariates were included in the primary analyses.

### Quality of Measurements

Two research assistants received approximately 10 hours of training before the study on how to rate each score in individuals with ASD based on behavioral indicators. Anxiety was evaluated using observable physical indicators, including gaze avoidance, behavioral freezing, reduced vocal volume, and repetitive movements. Self-confidence was primarily assessed based on the participants’ willingness to reattempt the task after failure and their ability to initiate interactions by proactively addressing others. Motivation was assessed by the participants’ persistence in continuing guiding behaviors and the presence or absence of avoidance behaviors. Metacognition was evaluated based on how the participants assessed and reflected on their performance. Self-efficacy was assessed by the participants’ ability to remain engaged as task difficulty increased and their capacity to verbalize which aspects of the task they found difficult. After the training, the research assistants completed practice ratings using sample cases to ensure consistent application of the rating criteria. They then independently rated each participant based on the predefined behavioral indicators. The 2 observers (ie, research assistants) demonstrated moderate-to-good interrater reliability. The intraclass correlation coefficient (ICC) values for anxiety, self-confidence, motivation, metacognition, and self-efficacy were 0.77, 0.83, 0.65, 0.59, and 0.80, respectively. The average score from the 2 observers was used as the observer-rated score.

### Statistical Analysis

Statistical analysis was performed using SPSS (version 27.0; IBM Corp). Descriptive statistics were used to analyze the data. Differences in age, estimated full IQ, AQ-J, each AASP domain, and LSAS scores between groups were analyzed using a 2-tailed *t* test. The difference in sex proportions was analyzed using the chi-square test. The overall study design involved 2 independent variables: group (TCR group vs control group) and time (1=baseline [day 0], 2=day 14, 3=day 28, 4=day 42, and 5=postintervention [day 56]). There were 10 independent variables: self-rated and observer-rated anxiety, self-confidence, motivation, metacognition, and self-efficacy related to social role–based behaviors. The study had 2 coprimary outcomes: self-rated and observer-rated self-efficacy scores related to social role–based behaviors, exactly as specified in the trial registry. Secondary outcomes included self-rated and observer-rated measures of anxiety, self-confidence, motivation, and metacognition related to social role–based behaviors. Baseline differences between groups in self-rated and observer-rated self-efficacy, anxiety, self-confidence, motivation, and metacognition related to social role–based behaviors were analyzed using a *t* test. To evaluate longitudinal changes in outcome measures between the TCR and control groups, a linear mixed model was used, including group (TCR vs control), time (days 0, 14, 28, 42, and 56), and group × time interaction as fixed effects. Time was treated as a categorical variable, and participant was included as a random effect.

The participants’ impressions of the appearance of the CG agent of AVACOM and the perceived usefulness of the experience for observing and evaluating the operator’s behavior were tested against the neutral midpoint (value 4) using the Wilcoxon signed-rank test. We used an α level of .05 for this complementary analysis. We applied the Bonferroni correction to control for the increased risk of type I error associated with multiple comparisons. Since we conducted 20 paired *t* tests across 10 outcome variables, the adjusted significance threshold was set at α=.05/20, which was approximately .0025. Given the exploratory nature and limited sample size, this approach may be conservative.

## Results

### Feasibility and Participation

We included 38 Japanese individuals with ASD. The TCR group included 20 participants (16 males) with a mean age of 25.6 (standard error of the mean [SEM] 1.2) years, a full-scale IQ of 88.5 (SEM 2.2), an AQ-J score of 32.4 (SEM 1.4), a low registration score of 38.8 (SEM 2.6), a sensation-seeking score of 38.4 (SEM 2.4), a sensory sensitivity score of 35.0 (SEM 2.4), a sensation-avoiding score of 38.2 (SEM 2.4), and an LSAS score of 62.6 (SEM 5.6). The control group included 18 participants (16 males) with a mean age of 25.5 (SEM 1.8) years, a full-scale IQ of 86.8 (SEM 3.2), an AQ-J score of 31.9 (SEM 1.4), a low registration score of 38.0 (SEM 1.8), a sensation-seeking score of 39.1 (SEM 2.8), a sensory sensitivity score of 36.4 (SEM 2.2), a sensation-avoiding score of 40.5 (SEM 1.7), and an LSAS score of 59.3 (SEM 7.9). There were no significant differences between the groups in terms of mean age (*P*=.98), sex proportion (*P*=.66), AQ-J score (*P*=.84), IQ (*P*=.66), low registration score (*P*=.82), sensation-seeking score (*P*=.84), sensory sensitivity score (*P*=.66), sensation-avoiding score (*P*=.44), or LSAS score (*P*=.73). The details are presented in Table 1.

**Table 1:** Descriptive characteristics of the TCR^a^ and control groups.

Characteristic		TCR group (n=20), mean (SEM^b^)	Control group (n=18), mean (SEM)	Chi-square (*df*) or *t* test (*df*)	*P* value
Age (years)		25.6 (1.2)	25.5 (1.8)	0.023 (36)^c^	.98
Sex (male: female), n		16:4	16:2	0.563 (1)^d^	.66
IQ		88.5 (2.2)	86.8 (3.2)	0.437 (36)^c^	.84
AQ-J^e^		32.4 (1.4)	31.9 (1.4)	0.208 (36)^c^	.66
AASP^f^					
	Low registration	38.8 (2.6)	38.0 (1.8)	0.233 (36)^c^	.82
	Sensation-seeking	38.4 (2.4)	36.4 (2.8)	−0.208 (36)^c^	.84
	Sensory sensitivity	35.0 (2.4)	36.4 (2.2)	−0.440 (36)^c^	.66
	Sensation-avoiding	38.2 (2.4)	40.5 (1.7)	−0.773 (36)^c^	.44
	LSAS^g^	62.6 (5.6)	58.3 (7.9)	0.344 (36)^c^	.73

^a^TCR: a training system that enables users to perform role-based behaviors using a computer graphics avatar in a real, bustling commercial facility.

^b^SEM: standard error of the mean.

^c^Represents a *t* test statistic.

^d^Represents a chi-square statistic.

^e^AQ-J: Autism-Spectrum Quotient–Japanese version; higher scores reflect a greater number of autism spectrum disorder–specific behaviors.

^f^AASP: Adolescent/Adult Sensory Profile.

^g^LSAS: Liebowitz Social Anxiety Scale.

The baseline means (SEM) for self-rated self-efficacy, anxiety, self-confidence, motivation, and metacognition in the TCR group were 5.80 (0.32), 2.80 (0.37), 3.85 (0.38), 3.25 (0.40), and 3.50 (0.39), respectively. The baseline means (SEM) for observer-rated self-efficacy, anxiety, self-confidence, motivation, and metacognition in the TCR group were 3.90 (SEM 0.25), 5.85 (SEM 0.30), 3.35 (SEM 0.31), 4.53 (SEM 0.19), and 3.93 (SEM 0.19), respectively. The baseline means (SEM) for self-rated self-efficacy, anxiety, self-confidence, motivation, and metacognition in the control group were 4.28 (SEM 0.46), 4.44 (SEM 0.44), 3.22 (SEM 0.45), 4.22 (SEM 0.47), and 4.28 (SEM 0.50), respectively. The baseline means (SEM) for observer-rated self-efficacy, anxiety, self-confidence, motivation, and metacognition in the control group were 4.03 (SEM 0.25), 4.89 (SEM 0.33), 3.39 (SEM 0.24), 4.14 (SEM 0.26), and 3.72 (SEM 0.24), respectively. There were no significant differences between the groups in terms of self-rated self-efficacy (*P*=.20), self-confidence (*P*=.24), motivation (*P*=.27), and metacognition (*P*=.12) or observer-rated self-efficacy (*P*=.72), self-confidence (*P*=.92), motivation (*P*=.23), and metacognition (*P*=.40). There were significant differences in self-rated anxiety (*P*=.02) and observer-rated anxiety (*P*=.04).

Each participant in the TCR and control groups attended 5 sessions. There were no missing data at either the participant level (0%) or the item level (0%) in this study. All enrolled participants (n=38) were included in the final analysis, and no attrition or loss to follow-up occurred. Therefore, all analyses were effectively conducted on the full sample, and no separate per-protocol analysis was required. All participants in the TCR group completed the trial procedure without any technological challenges or notable distress that could lead to session termination. Moreover, no incidents of confusion were observed during interactions between participants and unfamiliar people in a bustling commercial facility. No participant dropped out of the study. We carefully observed the participants’ performances through video recordings and confirmed with a trainer sitting next to the user that all participants remained highly motivated from start to finish. Two assessors independently reviewed the video recordings captured in front of the participants and confirmed that the participants communicated with unfamiliar individuals displayed on the PC without averting their gaze.

### Primary Analyses

For self-reported self-efficacy, the linear mixed model showed greater improvement from baseline in the TCR group than in the control group at day 42 (t_144.016_=2.206; *P*=.03; 95% CI 0.116-2.107) and day 56 (t_144.016_=2.670; *P*=.008; 95% CI 0.349-2.340). Although these effects did not remain significant after Bonferroni correction for multiple comparisons (adjusted significance threshold: *P*<.003), small-to-moderate effect sizes were observed (day 42: Cohen *d*=0.37; day 56: Cohen *d*=0.45).

For observer-reported self-efficacy, the linear mixed model showed greater improvement from baseline in the TCR group than in the control group at day 42 (t_144.016_=6.456; *P*<.001; Cohen *d*=1.08; 95% CI 0.719-1.353) and day 56 (t_144.016_=4.500; *P*<.001; Cohen *d*=0.75; 95% CI 0.405-1.039). These large effect sizes remained statistically significant after Bonferroni correction.

### Secondary Analyses

For self-reported anxiety, the linear mixed model showed greater reductions from baseline in the TCR group than in the control group at day 42 (t_144.016_=–2.243; *P*=.03; Cohen *d*=0.37; 95% CI –2.341 to –0.148) and day 56 (t_144.016_=–2.723; *P*=.007; Cohen *d*=0.45; 95% CI –2.608 to –0.414). These effect sizes were in the small-to-moderate range. However, these effects did not remain significant after Bonferroni correction for multiple comparisons (adjusted significance threshold: *P*<.003).

For observer-reported anxiety, the linear mixed model showed greater reductions from baseline in the TCR group than in the control group at day 42 (t_144.016_=–3.886; *P*=.001; Cohen *d*=0.65; 95% CI –1.500 to –0.489) and day 56 (t_144.016_=–3.593; *P*<.001; Cohen *d*=0.60; 95% CI –1.425 to –0.414).

For self-reported self-confidence, the linear mixed model showed greater improvement from baseline in the TCR group than in the control group at day 42 (t_144.016_=2.586; *P*=.011; Cohen *d*=0.43; 95% CI 0.279-2.088) and day 56 (t_144.016_=2.319; *P*=.02; Cohen *d*=0.39; 95% CI 0.157-1.965). These effect sizes were in the small-to-moderate range. However, these effects did not remain significant after Bonferroni correction for multiple comparisons (adjusted significance threshold: *P*<.003).

For observer-reported self-confidence, the linear mixed model showed greater improvement from baseline in the TCR group than in the control group at day 42 (t_144.016_=3.877; *P*<.001; Cohen *d*=0.65; 95% CI 0.380-1.170) and day 56 (t_144.016_=3.321; *P*=.001; Cohen *d*=0.55; 95% CI 0.269-1.059).

For self-reported motivation, the linear mixed model showed greater improvement from baseline in the TCR group than in the control group at day 42 (t_144.016_=2.522; *P*=.01; Cohen *d*=0.42; 95% CI 0.211-1.744). At day 56, the difference in change from baseline between groups did not reach statistical significance (t_144.016_=1.863; *P*=.06; Cohen *d*=0.31; 95% CI –0.044 to 1.489). These effect sizes were in the small-to-moderate range. However, these effects did not remain significant after Bonferroni correction for multiple comparisons (adjusted significance threshold: *P*<.003).

For observer-reported motivation, the linear mixed model showed greater improvement from baseline in the TCR group than in the control group at day 42 (t_144.016_=2.298; *P*=.02; Cohen *d*=0.38; 95% CI 0.067-0.894) and day 56 (t_144.016_=2.471; *P*=.02; Cohen *d*=0.41; 95% CI 0.103-0.930). These effect sizes were in the small-to-moderate range. However, these effects did not remain significant after Bonferroni correction for multiple comparisons (adjusted significance threshold: *P*<.003).

For self-reported metacognition, the linear mixed model showed greater improvement from baseline in the TCR group than in the control group at day 42 (t_144.016_=2.206; *P*=.03; Cohen *d*=0.37; 95% CI 0.116-2.107) and day 56 (t_144.016_=2.670; *P*=.008; Cohen *d*=0.45; 95% CI 0.349-2.340). These effect sizes were in the small-to-moderate range. However, these effects did not remain significant after Bonferroni correction for multiple comparisons (adjusted significance threshold: *P*<.003).

For observer-reported metacognition, the linear mixed model did not show significant differences in change from baseline between the TCR and control groups at day 42 (t_144.016_=1.815; *P*=.07; Cohen *d*=0.30; 95% CI –0.032 to 0.743) or day 56 (t_144.016_=1.858; *P*=.06; Cohen *d*=0.31; 95% CI –0.023 to 0.751). These effect sizes were in the small range. These effects did not reach statistical significance.

Tables 2 and 3 provide the details. The relationships between time and outcome scores are presented in Figures S1-S10 in Multimedia Appendix 1.

**Table 2:** Means and SEM^a^ for anxiety, self-confidence, motivation, metacognition, and self-efficacy scores in self-rated and observer-rated questionnaires 2 weeks before the intervention (day 0), immediately before the intervention (day 14), during the intervention (day 28), immediately after the intervention (day 42), and 2 weeks later after the intervention (day 56) in the TCR^b^ and control groups, and the interaction effects between the TCR and control groups in both questionnaire types.

Outcome and group		Day 0, mean (SEM)	Day 14, mean (SEM)	Day 28, mean (SEM)	Day 42, mean (SEM)	Day 56, mean (SEM)	Interaction effect			
							*F* statistic		*P* value	
Self-efficacy score in a self-rated questionnaire								2.886		.03
	TCR	3.50 (0.39)	3.60 (0.38)	4.25 (0.46)	4.25 (0.46)	4.50 (0.44)				
	Control	4.28 (0.46)	4.39 (0.45)	4.22 (0.48)	4.67 (0.46)	4.22 (0.42)				
Self-efficacy score in an observer-rated questionnaire								13.014		<.001
	TCR	3.90 (0.25)	4.40 (0.25)	4.95 (0.27)	5.33 (0.29)	5.15 (0.27)				
	Control	4.03 (0.25)	4.25 (0.23)	4.36 (0.22)	4.41 (0.21)	4.56 (0.22)				
Anxiety score in a self-rated questionnaire								3.009		.02
	TCR	5.80 (0.32)	5.35 (0.36)	4.70 (0.37)	4.50 (0.39)	4.40 (0.37)				
	Control	4.44 (0.44)	4.17 (0.48)	3.67 (0.45)	4.39 (0.46)	4.56 (0.43)				
Anxiety score in an observer-rated questionnaire								5.584		<.001
	TCR	5.85 (0.30)	5.33 (0.32)	4.80 (0.30)	4.30 (0.30)	4.38 (0.32)				
	Control	4.89 (0.33)	4.61 (0.30)	4.44 (0.28)	4.33 (0.28)	4.33 (0.34)				
Self-confidence score in a self-rated questionnaire								3.335		.01
	TCR	2.80 (0.37)	3.15 (0.41)	4.15 (0.47)	5.15 (0.33)	4.75 (0.29)				
	Control	3.22 (0.45)	3.67 (0.50)	3.89 (0.48)	4.39 (0.47)	4.11 (0.50)				
Self-confidence score in an observer-rated questionnaire								5.575		<.001
	TCR	3.35 (0.31)	3.73 (0.30)	4.33 (0.32)	4.63 (0.30)	4.38 (0.30)				
	Control	3.39 (0.24)	3.61 (0.22)	3.83 (0.21)	3.89 (0.20)	3.75 (0.23)				
Motivation score in a self-rated questionnaire								3.631		.008
	TCR	3.85 (0.37)	3.80 (0.37)	4.70 (0.34)	5.05 (0.37)	4.35 (0.39)				
	Control	4.22 (0.46)	4.22 (0.48)	4.06 (0.45)	4.44 (0.44)	4.00 (0.44)				
Motivation score in an observer-rated questionnaire								4.448		.002
	TCR	4.53 (0.19)	4.70 (0.20)	5.28 (0.20)	5.20 (0.20)	5.13 (0.21)				
	Control	4.14 (0.26)	4.22 (0.24)	4.14 (0.23)	4.33 (0.24)	4.22 (0.24)				
Metacognition score in a self-rated questionnaire								4.303		.003
	TCR	3.25 (0.40)	3.85 (0.40)	5.05 (0.29)	4.75 (0.41)	4.65 (0.40)				
	Control	4.28 (0.25)	4.22 (0.48)	4.11 (0.48)	4.67 (0.49)	4.33 (0.46)				
Metacognition score in an observer-rated questionnaire								3.815		<.006
	TCR	3.93 (0.19)	3.83 (0.22)	4.40 (0.15)	4.48 (0.19)	4.40 (0.17)				
	Control	3.72 (0.14)	3.78 (0.18)	3.72 (0.10)	3.92 (0.19)	3.83 (0.20)				

^a^SEM: standard error of the mean.

^b^TCR: a training system that enables users to perform role-based behaviors using a computer graphics avatar in a real, bustling commercial facility.

**Table 3:** Group × day interaction terms from the linear mixed models: between-group differences in change from baseline.

Outcome	Rater	Day	β (95% CI)	*t* statistic	Cohen *d*	*P* value
Self-efficacy	Self-report	42	1.11 (0.116 to 2.107)	2.206	0.37	.03
Self-efficacy	Self-report	56	1.34 (0.349 to 2.340)	2.670	0.45	.008
Self-efficacy	Observer	42	1.04 (0.719 to 1.353)	6.456	1.08	<.001
Self-efficacy	Observer	56	0.72 (0.405 to 1.039)	4.500	0.75	<.001
Anxiety	Self-report	42	–1.24 (–2.341 to –0.148)	–2.243	0.37	.03
Anxiety	Self-report	56	–1.51 (–2.608 to –0.414)	–2.723	0.45	.007
Anxiety	Observer	42	–0.99 (–1.500 to –0.489	–3.886	0.65	.001
Anxiety	Observer	56	–0.92 (–1.425 to –0.414)	–3.593	0.60	<.001
Self-confidence	Self-report	42	1.18 (0.279 to 2.088)	2.586	0.43	.01
Self-confidence	Self-report	56	1.06 (0.157 to 1.965)	2.319	0.39	.02
Self-confidence	Observer	42	0.78 (0.380 to 1.170)	3.877	0.65	<.001
Self-confidence	Observer	56	0.66 (0.269 to 1.059)	3.321	0.55	.001
Motivation	Self-report	42	0.98 (0.211 to 1.744)	2.522	0.42	.02
Motivation	Self-report	56	0.72 (–0.044 to 1.489)	1.863	0.31	.06
Motivation	Observer	42	0.48 (0.067 to 0.894)	2.298	0.38	.02
Motivation	Observer	56	0.52 (0.103 to 0.930)	2.471	0.41	.02
Metacognition	Self-report	42	1.11 (0.116 to 2.107)	2.206	0.37	.03
Metacognition	Self-report	56	1.34 (0.349 to 2.340)	2.670	0.45	.008
Metacognition	Observer	42	0.36 (–0.032 to 0.743)	1.815	0.30	.07
Metacognition	Observer	56	0.36 (–0.023 to 0.751)	1.858	0.31	.06

The interventions were conducted once per week for 5 weeks (days 14, 21, 28, 35, and 42). Anxiety, self-confidence, motivation, metacognition, and self-efficacy related to performing social role–based behaviors were assessed in all participants in the TCR and control groups 2 weeks before the intervention (day 0), immediately before the intervention (day 14), during the intervention (day 28), immediately after the intervention (day 42), and 2 weeks after the intervention (day 56) by the participants themselves and by 2 research assistants who were unaware of the study’s objectives. The average score from the 2 observers was used as the observer-rated score.

β values represent the estimated group × day interaction coefficients (between-group differences in change from baseline) derived from the linear mixed models. Positive values indicate greater increases in the TCR group relative to the control group, whereas negative values indicate greater reductions in the TCR group relative to the control group. Reported *t* values, *P* values, CIs, and effect sizes correspond to the estimated differences in change from baseline between the TCR and control groups at each time point.

A Wilcoxon signed-rank test against the neutral value of 4 indicated that participants rated the appearance of the CG agent of AVACOM as socially desirable (*z*=13.00; *P*=.007) and perceived the experience of observing and evaluating the operator’s behavior as useful (*z*=13.50; *P*=.004).

## Discussion

### Principal Findings

This study evaluated the feasibility and potential effects of TCR, a training system that enables individuals with ASD to perform social role–based behaviors using a CG avatar in a realistic environment. Overall, the findings suggest that TCR was feasible to implement and may contribute to improvements in social role–based behaviors. Participants who received TCR in addition to CGT generally demonstrated reduced anxiety and enhanced self-confidence, motivation, metacognition, and self-efficacy compared with those who received CGT alone. These findings suggest that TCR may support the development of functional social skills and facilitate their application in real-world contexts.

When comparing preintervention and postintervention outcomes, similar patterns were observed across self-reported and observer-rated questionnaire responses, with the exception of self-reported motivation and self-efficacy at specific time points. This general consistency supports the validity of the observed changes and suggests that the improvements were not solely attributable to a subjective response bias. In contrast to previous reports suggesting that improving self-efficacy in individuals with ASD is challenging [52], the present findings indicate that TCR may contribute to enhanced self-efficacy for social role–based behaviors. This finding is particularly noteworthy because self-efficacy is considered a key determinant of whether individuals initiate, continue, and successfully perform challenging social behaviors [8].

One possible explanation for these findings is that TCR integrates multiple sources of self-efficacy as conceptualized in social learning theory by Bandura [9], including mastery experiences, vicarious experiences, verbal persuasion, and physiological and affective states, along with a gradual increase in levels of challenge, clear feedback, and peer support. Moreover, the results from the self-questionnaire regarding the evaluation of the operator’s behavior suggest that the experience of observing and evaluating the operator’s behavior is beneficial for participants. These findings suggest that a multifaceted training approach may be effective in enhancing the self-efficacy of individuals with ASD.

A growing body of literature indicates that many individuals with ASD desire and are motivated to use technology [53]. Similar improvements in engagement have been reported in previous technology-based interventions for ASD [17]. The technology behind TCR may increase users’ enthusiasm for and concentration on the program [54]. The Proteus effect is the tendency of individuals to be affected by their digital representations, such as avatars [55]. Our results suggest that the participants’ impressions of the appearance of the CG agent of AVACOM were perceived as socially desirable. Previous studies have suggested that avatar-mediated communication may reduce social anxiety and facilitate self-expression [37]. It is possible that the CG agent of AVACOM, designed with an inherently expressive face and communicative appearance, helped reduce anxiety during interactions with unfamiliar individuals, thereby facilitating more active engagement in social role–based behavior [56].

Although individuals with ASD may express an interest in novel technologies [57], they may also experience significant cognitive overload when using complex digital systems [58]. In this study, the TCR system was designed to be easy to use even for participants with limited digital skills. Notably, all participants in the TCR group completed the trial procedures without experiencing technological difficulties or distress that would have required session termination. The ease of use of the system across users with diverse digital skill levels may have partially contributed to the high completion rates observed in this study [59].

### Generalizability

Individuals with ASD often exhibit poor generalization to novel postintervention settings [60-64]. The findings indicated that while some interventions led to initial improvements, there was a marked decrease in effectiveness when these skills were applied to everyday situations [58,64]. Previous studies have emphasized that interventions conducted in ecologically valid settings are more likely to promote skill generalization [37]. In TCR, individuals with ASD communicated with unfamiliar people in real-life settings using the CG agent of AVACOM. In this setting, they did not need to apply the learned skills across different scenarios, which may be one reason for attaining generalizability in TCR [65].

### Implications

Based on our findings and implementation experience, we propose several considerations for future interventions: (1) use of a CG avatar in a real-world setting, (2) provision of appropriately graded challenges to support skill acquisition, (3) incorporation of peer support to facilitate engagement, and (4) use of structured feedback to reinforce behavioral change. These design elements may be particularly important for interventions targeting social role–based behaviors, as they provide opportunities for repeated practice, observation, and reinforcement in ecologically valid settings. These findings support the potential integration of TCR into community-based rehabilitation programs aimed at improving social participation in adults with ASD. Such programs may also facilitate the development of communication and role-based skills relevant to employment, community participation, and independent living. Because the intervention can be delivered in naturalistic community environments, TCR may be readily incorporated into existing psychosocial rehabilitation services with relatively limited additional resources. Moreover, TCR may provide a practical approach for clinicians and community support providers seeking to improve social communication and role-based skills in naturalistic settings. The usefulness of operating an avatar has also been suggested for individuals with social anxiety disorder [36] and schizophrenia [66,67] who often struggle to communicate with unfamiliar people. Therefore, TCR may be beneficial for improving communication skills in individuals living with these disorders. Further research is required to explore the applicability of this approach under other conditions.

### Limitations

This study has some limitations. Given that the estimated required sample size exceeded 70 participants, the present study had a relatively small sample size. The relatively wide CIs suggest limited precision of the estimates, likely reflecting the small sample size of this pilot study. Although the sex ratio [68] and IQ [69] distribution in our sample were generally consistent with those reported in previous studies of adults with ASD, the participants may not fully represent the broader ASD population. The application of a conservative significance level resulted in outcomes that did not reach statistical significance. Although several outcomes did not reach statistical significance under the conservative significance threshold, the observed patterns were generally consistent with potential improvements in anxiety, self-confidence, motivation, metacognition, and self-efficacy. Despite these limitations, these findings indicate that self-reported and observed changes may have meaningful clinical implications for improving social role–based behaviors in individuals with ASD. Future studies with larger sample sizes and more female participants are necessary to evaluate the feasibility of TCR in individuals with ASD. Second, potential bias due to the nonrandomized allocation of participants, as well as the lack of blinding, may be additional limitations of this study. Third, although previous studies [70,71] have shown the potential of DISCO as a valid and reliable instrument, a gold-standard measure (Autism Diagnostic Observation Schedule [72] and Autism Diagnostic Interview–Revised [73]) should have been considered. Future studies are recommended to use gold-standard measures to overcome these limitations. Fourth, the control group received only controlled communication guidance from teachers in a classroom setting. Therefore, it is not possible to determine whether a communication training system in virtual reality, which is less resource-intensive than using a CG avatar on real city streets, would be as effective. Fifth, the study was registered retrospectively, as the registration was completed after the start of participant enrollment. This may affect the transparency of the study protocol. Sixth, each outcome was assessed using a single-item, nonvalidated measure, which may introduce measurement bias and limit the reliability and validity of the findings. These measures were selected to reduce participant burden in this feasibility study and to facilitate repeated assessments in a real-world setting. However, future studies should use standardized and validated multiitem instruments to confirm these results.

### Conclusion

This study is innovative in that TCR enables users to perform social role–based behaviors, such as guiding and assisting unfamiliar people, in a bustling commercial environment while remaining in a safe and familiar physical setting. Unlike that in the previous study [37], TCR uniquely integrates peer observation, role modeling, and real-time trainer support, thereby incorporating multiple sources of self-efficacy, including vicarious experiences, verbal persuasion, and structured feedback. By demonstrating the feasibility and potential of TCR in enhancing self-efficacy for social role–based behaviors, this study contributes to advancing intervention strategies for adults with ASD. More broadly, these findings highlight the potential of avatar-mediated interventions to bridge the gap between structured training environments and real-world social participation, an issue that has long challenged psychosocial interventions for individuals with ASD. Future studies with larger and more diverse samples are warranted to extend these findings.

## Data Availability

The datasets generated and analyzed during this study are available from the corresponding author on reasonable request.

## Appendix

Multimedia Appendix 1 Supplementary figures.

## Abbreviations

AASP: Adolescent/Adult Sensory Profile
ADHD: attention-deficit/hyperactivity disorder
AQ-J: Autism-Spectrum Quotient–Japanese version
ASD: autism spectrum disorder
CG: computer graphics
CGT: communication guidance from teachers
DISCO: Diagnostic Interview for Social and Communication Disorders
DSM-5: Diagnostic and Statistical Manual of Mental Disorders (Fifth Edition)
ICC: intraclass correlation coefficient
JART: Japanese Adult Reading Test
LSAS: Liebowitz Social Anxiety Scale
SEM: standard error of the mean
TCR: a training system that enables users to perform role-based behaviors using a computer graphics avatar in a real, bustling commercial facility
VR: virtual reality
