# Supplementary material for: Social Role–Based Training Using a Computer Graphics Avatar in a Real-World Commercial Setting for Individuals With Autism Spectrum Disorder: Quasi-Experimental Study
Source: J Med Internet Res. 2026 Sep 30;28:e93549. doi: 10.2196/93549 (PMC13669708; doi:10.2196/93549)
Supplement: Supplementary file 1 [file jmir_v28i1e93549_app1.docx]

**Figure S1.** Mean self-rated anxiety scores related to performing social role-based behaviours in real-world interactions in the TCR group and the control group across five time points (Day 0, Day 14, Day 28, Day 42 and Day 56).

**Figure S2.** Mean observer-rated anxiety scores related to performing social role-based behaviours in real-world interactions in the TCR group and the control group across five time points (Day 0, Day 14, Day 28, Day 42 and Day 56).

**Figure S3.** Mean self-rated self-confidence scores related to performing social role-based behaviours in real-world interactions in the TCR group and the control group across five time points (Day 0, Day 14, Day 28, Day 42 and Day 56).

**Figure S4.** Mean observer-rated self-confidence scores related to performing social role-based behaviours in real-world interactions in the TCR group and the control group across five time points (Day 0, Day 14, Day 28, Day 42 and Day 56).

**Figure S5.** Mean self-rated motivation scores related to performing social role-based behaviours in real-world interactions in the TCR group and the control group across five time points (Day 0, Day 14, Day 28, Day 42 and Day 56).

**Figure S6.** Mean observer-rated motivation scores related to performing social role-based behaviours in real-world interactions in the TCR group and the control group across five time points (Day 0, Day 14, Day 28, Day 42 and Day 56).

**Figure S7.** Mean metacognition scores related to performing social role-based behaviours in real-world interactions in the TCR group and the control group across five time points (Day 0, Day 14, Day 28, Day 42 and Day 56).

**Figure S8.** Mean observer-rated metacognition scores related to performing social role-based behaviours in real-world interactions in the TCR group and the control group across five time points (Day 0, Day 14, Day 28, Day 42 and Day 56).

**Figure S9.** Mean self-efficasy scores related to performing social role-based behaviours in real-world interactions in the TCR group and the control group across five time points (Day 0, Day 14, Day 28, Day 42 and Day 56).

**Figure S10.** Mean observer-rated metacognition scores related to performing social role-based behaviours in real-world interactions in the TCR group and the control group across five time points (Day 0, Day 14, Day 28, Day 42 and Day 56).
